# Comparative Study of 5-Day and 10-Day Cefditoren Pivoxil Treatments for Recurrent Group A *β*-Hemolytic *Streptococcus pharyngitis* in Children

**DOI:** 10.1155/2009/863608

**Published:** 2009-03-05

**Authors:** Hideaki Kikuta, Mutsuo Shibata, Shuji Nakata, Tatsuru Yamanaka, Hiroshi Sakata, Kouji Akizawa, Kunihiko Kobayashi

**Affiliations:** Pediatric Clinic, Touei Hospital, N-41, E-16, Higashi-ku, Sapporo 007-0841, Japan

## Abstract

Efficacy of short-course therapy with cephalosporins for treatment of group A *β*-hemolytic streptococcus (GABHS) pharyngitis is still controversial. Subjects were 226 children with a history of at least one episode of GABHS pharyngitis. Recurrence within the follow-up period (3 weeks after initiation of therapy) occurred in 7 of the 77 children in the 5-day treatment group and in 1 of the 149 children in the 10-day treatment group; the incidence of recurrence being significantly higher in the 5-day treatment group. Bacteriologic treatment failure (GABHS isolation without overt pharyngitis) at follow-up culture was observed in 7 of the 77 children in the 5-day treatment group and 17 of the 149 children in the 10-day treatment group. There was no statistical difference between the two groups. A 5-day course of oral cephalosporins is not always recommended for treatment of GABHS pharyngitis in children who have repeated episodes of pharyngitis.

## 1. Introduction

Group A *β*-hemolytic streptococci (GABHS, also referred to as *Streptococcus pyogenes*) are the most common bacterial cause of acute pharyngitis, accounting for approximately 15 to 30% of cases in children and 5 to 10% of cases in adults [1–3]. The objectives of treatment for GABHS pharyngitis are prevention of rheumatic fever, prevention of suppurative complications, reduction in transmission of GABHS, and shortening of the clinical course. Although the standard oral recommended therapy for GABHS pharyngitis has long been penicillin V for 10 days, there has been no change in the susceptibility of GABHS for over 50 years [1–4]. However, recurrent GABHS infections occurred more frequently in the 1990s than the 1970s, and treatment failure rates as high as 10 to 30% have been reported [5, 6]. Reasons for failure of the standard therapy include an increase in *β*-lactamase-producing pharyngeal pathogens that inactivate penicillin, poor compliance, intracellular location of GABHS, eradication of protective pharyngeal *α*-streptococci, and increased exposure in crowded day-care centers and schools [7–9].

The increase in failure rate of the standard 10-day course of therapy with penicillin has heightened interest in alternative treatments for this infection. Cephalosporins have been used successfully for treatment of this infection since the 1970s [10, 11]. A meta-analysis showed that 10-day cephalosporin treatment was superior in eradication of GABHS and in clinical response [12]. Recent studies on the efficacy of a short-course treatment (5 days) with cephalosporins compared with that of 10-day penicillin V treatment have shown that the shorter cephalosporin treatments were equivalent or superior in eradication of GABHS and in clinical response [13]. However, there are several controversial issues regarding the increase in recurrent GABHS pharyngitis since the 1990s, reasons for failure of the standard penicillin therapy and evaluation of alternative cephalosporin treatments for GABHS pharyngitis [14–16]. The purpose of this study was to determine the efficacy of a 5-day course of oral cephalosporin compared with that of a 10-day course of oral cephalosporin for recurrent GABHS pharyngitis in children.

## 2. Materials and Methods

### 2.1. Subjects

In a prospective, multicenter surveillance study, a total of 299 children with a history of at least 1 episode of GABHS pharyngitis were enrolled at private offices of 41 practicing pediatricians and hospitals of 23 practicing pediatricians between May 2006 and August 2007 in Hokkaido, the northernmost island of Japan. A total of 73 children were excluded after enrollment because of incomplete follow-up (*n* = 61), inappropriate enrollment (*n* = 11), and noncompliance (*n* = 1). The number of eligible patients for the study was 226. The 226 children (138 males and 88 females; 2 to 14 years old; mean age: 6.8 years; SD: 2.0 years) were diagnosed as having GABHS pharingitis by both clinical findings and a positive reaction in pharyngeal swab specimens using commercially available rapid antigen detection tests (RADTs) for GABHS (Immunocard ST Strep A, TFB, INC., Tokyo; Dipstick Eiken Strept A, Eiken Chemical Co., Ltd., Tokyo; Quickview Strep A, Wako Pure Chemical Industries, Ltd., Osaka). The clinical findings included presence of oropharyngeal erythema or tonsillar exudate, fever, cervical lymphadenitis, and sore throat. Patients with signs and symptoms suggestive of viral infection (rhinorrhea, cough, conjunctivitis) were excluded. Follow-up visit and follow-up culture were scheduled 3 weeks after the initiation of therapy. The culture samples were obtained by rubbing over the posterior portion of the pharynx and both tonsils with a sterile, rayon-tipped swab. All pharyngeal swab specimens were collected after obtaining informed consent from the patients or children's parents. The swabs were placed in an aerobic transport system and then transported to our laboratory. The children with subsequent GABHS pharyngitis within the follow-up period were also diagnosed by both clinical findings and results of RADTs. The 226 patients were treated with cefditoren pivoxil (9 mg/kg; maximum dose of 300 mg/day, 3 times a day) for 5 days (77 patients) or for 10 days (149 patients). Since each pediatrician arbitrarily decided the period of antibiotic treatment, there was no complete randomization of the patients.

### 2.2. Definition

Clinical response to treatment was defined here as recurrence (clinical treatment failure; clinically and RADT-confirmed GABHS pharyngitis within the follow-up period, irrespective of T serotype) and as clinical cure (clinical treatment success; no clinical findings of GABHS pharyngitis within the follow-up period). Bacteriologic response to treatment was defined here as eradication (bacteriologic treatment success; no GABHS isolation at follow-up visit) and as noneradication (bacteriologic treatment failure; GABHS isolation without overt pharyngitis at follow-up visit, irrespective of T serotype). Treatment success was, therefore, defined as bacteriologic treatment success with no recurrence, and treatment failure was defined as either clinical or bacteriologic treatment failure.

### 2.3. Serotyping

Serotyping was examined for T types by the agglutination method using Group A streptococcus hemolyticus T typing immunoserum “SEIKEN” (Denka Seiken Co., Ltd., Tokyo).

### 2.4. Statistical Analysis

Quantitative variables were compared using Welch's *t*-test, and qualitative variables were compared using the chi-square test with Fisher's exact test. For all statistical comparisons, *P* < .05 was considered to be significant.

## 3. Results and Discussion

### 3.1. Results

The characteristics of children with repeated GABHA pharyngitis are shown in Table 1. The mean number of episodes of repeated GABHS pharyngitis including the present episode was 2.79 (range: 2–12; SD: 1.35). Of the 226 patients, 182 (80.5%) patients had a history of at least 2 episodes of repeated GABHS pharyngitis. The mean interval between preceding and present episodes was 10.48 months (range: 0.4–79 months; SD: 13.85). Of the 226 patients, 98 (43.3%) patients had preceding GABHS pharyngitis within 3 months before the present GABHS pharyngitis. The mean length of follow-up excluding recurrence cases (definition of recurrence case given below) was 22.20 days (range: 14–35 days; SD: 2.98 days). Of the 226 patients, 218 (96.4%) patients were followed up for 14–28 days after the initiation of therapy. The characteristics of children treated for 5 days and 10 days are shown in Table 1. The ratios of males to females were 1 : 0.67 in the 5-day treatment group and 1 : 0.62 in the 10-day treatment group. Mean ages were 6.70 years (range: 3–12 years; SD: 1.93 years) in the 5-day treatment group and 6.80 years (range: 2–14 years; SD: 2.10 years) in the 10-day treatment group. Mean numbers of episodes of repeated GABHS pharyngitis were 2.75 (range: 2–12; SD: 1.54) in the 5-day treatment group and 2.81 (range: 2–8; SD: 1.24) in the 10-day treatment group. Mean intervals between preceding and present episodes were 12.95 months (range: 0.5–79 months; SD: 17.30 months) in the 5-day treatment group and 9.20 months (range: 0.4–55 months; SD: 11.47 months) in the 10-day treatment group. Mean lengths of follow-up excluding recurrence cases were 22.57 days (range: 14–32 days; SD: 3.03 days) in the 5-day treatment group and 22.02 days (range: 15–35 days; SD: 2.94 days) in the 10-day treatment group. There were no significant differences between the two treatment groups in the distribution of age, gender, number of episodes of repeated GABHS pharyngitis, interval between preceding and present episodes, and length of follow-up.

The clinical and bacteriological results are shown in Table 2. Recurrence (clinical treatment failure) occurred in 7 (9.1%) of the 77 children in the 5-day treatment group and in 1 (0.7%) of the 149 children in the 10-day treatment group. The 5-day treatment group showed a significantly higher incidence of recurrent GABHS pharyngitis than that in the 10-day treatment group (*P* = .003). The eight patients with recurrent GABHS phayryngitis included 5 males and 3 females with a mean age of 6.36 years (range: 5–8 years; SD: 0.91 years). The mean interval from onset of the index infection to onset of subsequent recurrent GABHS pharyngitis was 11.3 days after the initiation of therapy (range: 6–22 days; SD: 4.94 days). The mean number of episodes of repeated GABHS pharyngitis was 2.88 (range: 2–6; SD: 1.36). The mean interval between preceding and present episodes was 10.81 months (range: 0.5–24 months; SD: 8.268 months). There was no recognizable risk factor of recurrent GABHS pharyngitis in the patients, probably due to the limited number of recurrent cases. Bacteriologic treatment failure at the follow-up visit was observed in 7 (9.1%) of the 77 children in 5-day treatment group and in 17 (11.4%) of the 149 children in the 10-day treatment group. There was no significant difference between the two treatment groups (*P* = .655). The treatment failure rates were 18.2% in the 5-day group and 12.1% in the 10-day group. This difference was also not statistically significant (*P* = .231). No poststreptococcal sequelae were noted in this study.

The characteristics of children in the treatment success group (bacteriologic treatment success with no recurrence) and treatment failure group (either clinical or bacteriologic treatment failure) are shown in Table 3. Ratios of males to females were 1 : 0.73 in the treatment success group (194 children) and 1 : 0.23 in the treatment failure group (32 children). Treatment failure rates were significantly affected by gender (*P* = .011). Mean ages were 6.86 years (range: 2–14 years; SD: 2.12 years) in the treatment success group and 6.24 years (range: 3–9 years; SD: 1.39 years) in the treatment failure group (*P* = .036). Treatment failure was observed more frequently in the 86 children aged 6 to 7 years (in 22 of the 86 children, treatment failure rate of 25.6%) than in the 66 children aged 2 to 5 years (in 6 of the 66 children, treatment failure rate of 9.1%; *P* < .001) and in the 74 children aged 8 to 14 years (in 4 of the 74 children, treatment failure rate of 5.4%; *P* < .001). The mean number of episodes of repeated GABHS pharyngitis in the treatment success group was 2.77 (range: 2–6; SD: 1.36) and that in the treatment failure group was 2.91 (range: 2–12; SD: 1.26), the difference between the groups not being statistically significant (*P* = .586). The mean intervals between preceding and present episodes were 11.04 months (range: 0.5–79 months; SD: 14.57 months) in the treatment success group and 7.05 months (range: 0.4–27 months; SD: 7.31 months) in the treatment failure group. Although the difference between the intervals in the two groups was statistically significant (*P* = .019), the interval responsible for treatment failure could not be statistically determined.

All corresponding strains isolated from the 24 children with positive follow-up cultures both at pre- and posttreatments had the same serotype. Six different T types were identified, of which T12 was the most common serotype. In the 8 children with recurrence within the follow-up period, only 4 corresponding strains isolated at pretreatment and at recurrence were available for characterization by serotyping. GABHS with different serotype was identified in one child in the 5-day treatment group but not in other children.

### 3.2. Discussion

Recurrence of GABHS pharyngitis frequently occurs in children after treatment. Approximately 10 to 20% of children have recurrent infection within 1 year of an index infection [5, 11, 17]. Possible explanations for the recurrence are 1) reinfection: new infection with GABHS, and 2) relapse: recurrence of originally persisted GABHS. Although serotyping of GABHS isolates helps to make the distinction, it may be difficult to distinguish between reinfection and relapse. Holm et al. reported that 10-day cephalosporin treatment in children with recurrent GABHS pharyngitis resulted in bacteriologic and clinical cure rates higher than those of 10-day penicillin treatment [18].

In this study, the recurrence rate within 3 weeks after the initiation of therapy was significantly higher in the 5-day treatment group than in the 10-day treatment group. However, the difference between the two treatment groups in rate of bacteriologic treatment failure at the follow-up visit was not statistically significant. All corresponding strains isolated at pre- and posttreatments in the 24 patients with bacteriologic treatment failure had the same serotype, indicating that the treatment failure might be relapse. Only one of the corresponding isolates in 4 children with recurrence had different serotypes, indicating that the treatment failure in this case was reinfection. Test-of-cure timing, follow-up visit in this study, is important in the interpretation of treatment failure rate. It has been suggested that the test-of-cure evaluation should optimally be done within 3 to 14 days after completion of therapy [12]. Although we were not able to rule out the possibility of reinfection with a new strain, the fact that 96% of corresponding isolates during 3-week period had the same serotype in this study suggested that most of the treatment failure cases within 3 weeks were caused by relapse. Age of 6 to 7 years for boys and short interval between preceding and present episodes were associated with a significantly higher risk of treatment failure including recurrence and bacteriologic treatment failure. The frequency of episodes of recurrent GABHS pharyngitis had no effect on treatment failure.

In comparative GABHS pharyngitis antibiotic trials, it is difficult to distinguish between a GABHS carrier with an intercurrent viral infection and a patient with acute GABHS pharyngitis. Penicillin is not so effective in eradication of GABHS in carriers, whereas cephalosporins are effective [18]. The incidence of GABHS carriers is approximately 5 to 20% [19]. In our study, GAS carriers, if any, would have been equally distributed in the two treatment groups. Furthermore, to reduce the risk of enrolling GABHS carriers with concurrent viral pharyngitis, we excluded patients with symptoms such as rhinorrhea, cough, and conjunctivitis.

The results of a previous meta-analysis indicated that 5-day or 10-day administration cephalosporins were superior to 10-day administration penicillin for treatment of GABHS pharyngitis although the results are controversial [14–16]. There was no significant difference between treatment failures at the follow-up visit in the cephalosporin treatment groups in our study. However, subsequent GABHS pharyngitis within the follow-up period occurred more frequently in the 5-day treatment group than in the 10-day treatment group.

## 4. Conclusion

A 5-day course of oral cephalosporins is not always recommended for treatment of recurrent GABHS pharyngitis in children, especially for 6- to 7-year-old boys and for children with a short interval between preceding and present episodes. Sincethis study has a limitation because of its nonrandomized design, further studies are necessary to determine how best to reduce treatment failure for recurrent GABHS pharyngitis in children.

## Figures and Tables

**Table 1:** Characteristics of children with repeated GABHA pharyngitis.

Characteristics	Total	5-day treatment group	10-day treatment group	*P* value
Number of cases	226	77	149	
Male:Female	138 : 88	46 : 31	92 : 57	.775
Age (years) (mean±SD)	6.77 ± 2.04	6.70 ± 1.93	6.80 ± 2.10	.728
Frequency of episodes (times) (mean±SD)	2.79 ± 1.35	2.75 ± 1.54	2.81 ± 1.24	.768
Interval between preceding and present episodes (months) (mean±SD)	10.48 ± 13.8	12.95 ± 17.30	9.20 ± 11.47	.089
Length of follow-up (days) (mean±SD)	22.20 ± 2.98	22.57 ± 3.03	22.02 ± 2.94	.203

**Table 2:** Clinical and bacteriological results.

Results	Total	5-day treatment group	10-day treatment group	*P* value
Recurrence (clinical treatment failure)	8/226	7/77	1/149	.003
Bacteriologic treatment failure (noneradication)	24/226	7/77	17/149	.655
Treatment failure	32/226	14/77	18/149	.231

**Table 3:** Characteristics of children with treatment success and treatment failure.

Characteristics	Treatment success group	Treatment failure group	*P* value
Number of cases	194	32	
Male:Female	112 : 82	26 : 6	.011
Age (years) (mean±SD)	6.86 ± 2.12	6.24 ± 1.39	.036
Frequency of episodes (times) (mean±SD)	2.77 ± 1.36	2.91 ± 1.26	.586
Interval between preceding and present episodes (months) (mean±SD)	11.04 ± 14.57	7.05 ± 7.31	.018
